# Antioxidants and Polyphenols: Concentrations and Relation to Male Infertility and Treatment Success

**DOI:** 10.1155/2016/9140925

**Published:** 2016-05-12

**Authors:** Tali Silberstein, Iris Har-Vardi, Avi Harlev, Michael Friger, Batel Hamou, Tamar Barac, Eli Levitas, Oshra Saphier

**Affiliations:** ^1^Department of Obstetrics and Gynecology, Soroka Medical Center, Ben-Gurion University of the Negev, Beersheba, Israel; ^2^Department of Public Health, Ben-Gurion University of the Negev, Beersheba, Israel; ^3^Department of Chemical Engineering, Sami Shamoon College of Engineering, Beersheba, Israel

## Abstract

Oxidative stress is induced by reactive oxygen substances (ROS) that are known to affect male fertility. The aims of this study were to prospectively investigate and characterize total antioxidant and specifically polyphenols concentrations and their relations to sperm quality and fertility treatment success. During their infertility treatment, sixty-seven males were prospectively recruited to this study. After separation of the sperm from the semen sample, the semen fluid samples antioxidants and polyphenols concentrations were determined. Antioxidant concentration was significantly associated with sperm concentration and total motile count. Antioxidants concentration in the group of male with sperm concentration ≥ 15 × 10^6^ was significantly higher than in the group of male with antioxidants concentration < 15 × 10^6^ (830.3 ± 350 *μ*M and 268.3 ± 220 *μ*M, resp., *p* < 0.001). Polyphenols concentration did not differ between the groups of sperm concentration above and below 15 × 10^6^ (178.7 ± 121 *μ*M and 161.7 ± 61 *μ*M, resp., *p*-NS). No difference was found between fertilization rates and antioxidants or polyphenols concentrations. This is the first study that reports on polyphenols concentration within semen fluid. More studies are needed in order to investigate polyphenols role in male fertility.

## 1. Background

Changes in human diets over the past 100 to 150 years, particularly in terms of dietary fat intake and total antioxidants, and its effect on human health, have become a major interest in nutrition research. Oxidative stress is induced by reactive oxygen substances (ROS), such as superoxide ^•^O_2_^−^, hydrogen peroxide H_2_O_2_, and hydroxyl radical ^•^OH, as well as reactive nitrogen substances (RNS) such as ^•^NO. These oxidants are formed during physiological processes or under the influence of chemical substances that cause damage to biological systems. The imbalance between the production of reactive oxidizing agents and a biological system's ability to readily detoxify the reactive intermediates or repair the consequential damage results in oxidative stress [1, 2]. ROS are free radicals that are involved in numerous sperm physiological processes such as capacitation, hyper activation, and sperm-oocyte fusion [3–6]. Oxidative stress has been shown to affect male fertility, as it can be seen in semen parameters and fertilizing measurements. Reduced fertility outcome was found to be associated with free radicals [7, 8]. In order to address the oxidative stress in the testis, it has antioxidant systems comprising both enzymes and free radical scavengers [9]. Usually, there is a balance between concentrations of reactive oxygen species and antioxidant scavenging systems that enable normal sperm production [10].

Polyphenols represent a group of chemical substances that is common in plants and is structurally characterized by the presence of one or more phenol units. Polyphenols are one of the most abundant antioxidants in human diet [1, 11]. In the literature, there is no report regarding polyphenols concentration in semen fluid. Since human diet is rich in the antioxidants polyphenols, it is logical to expect the polyphenols to be one of the antioxidants within semen fluid.

The aims of this study were as follows: (1) to determine antioxidants and specifically polyphenols concentrations within semen fluid, (2) to evaluate the relation between antioxidants and polyphenols concentrations in male semen fluid and sperm parameters, and (3) to evaluate antioxidants and polyphenols concentrations correlation to infertility treatment results.

The protocol for this study was approved by the Research Ethics Committee of Soroka University Medical Center. All participants were enrolled after signing informed consent.

## 2. Methods

### 2.1. Study Population and Semen Analysis

During a 6-month period, 67 patients were recruited to this prospective study. All patients were treated at the IVF clinic at the Soroka University Medical Center. Semen analysis was performed according to the published guidelines of WHO [12] and the routinely applied techniques in our IVF laboratory. Samples were collected by masturbation after 2-3 days of sexual abstinence. Routine sperm analysis and sperm preparation were initiated after liquefaction at RT (23°C) and within 1.5 h of production. The semen samples were separated from the sperm cells using density gradient or in cases of <1 × 10^6^/mL using centrifugation. A two-layer isolate gradient (80%, 40%) was prepared (Irvine Scientific, Santa Ana, CA, USA); the 80% gradient layer of 1.0 mL was layered with 1 mL of 40% and liquefied semen on top. This was centrifuged for 20 min at 310 g. The supernatant without gradient's elements was carefully collected and frozen in liquid nitrogen for later analysis. Spermatozoa were removed from the base of the 80% gradient layer and used for fertilization. In the severe OTA samples, we diluted the semen 1 : 1 with prewarmed (37°C) Quinn's Sperm Wash Medium (Sage Media, Trumbull, CT, USA) and transferred into a conical centrifuge tube (Becton Dickinson, NJ, USA) and centrifuged at 300 g for 10 minutes. The supernatant was carefully collected and frozen in liquid nitrogen for later analysis.

Sperm concentration and motility were assessed under the light microscope (Zeiss, Goettingen, Germany) at 200x magnification using Makler's counting chamber (0.01 mm^2^ and 10 *μ*m deep). The assessment was done immediately after sperm liquefaction and after each spermatozoa preparation technique.

### 2.2. Determination of Total Polyphenols Content

The total phenolic content in the seminal fluid was determined by using the Folin–Ciocalteu method [13]. This method is based on the redox reaction of the reagent, forming a blue color pigment, with typical absorbance at 760 nm. All UV-Vis measurements were performed using a Varian Cary Bio 100 UV-Vis spectrophotometer. An aliquot of 200 *μ*L of seminal fluid was mixed with 0.25 mL of Folin reagent and 0.5 mL saturated solution of sodium carbonate. This solution was vortexed for 15 seconds and was kept in dark for 30 minutes for color development. Then, the solution was centrifuged (5,300 rpm) for 10 minutes until it was transparent. The supernatant was collected and the solution absorbance was measured by using UV-Vis spectrophotometer at wavelength of 760 nm. The total amount of polyphenols was expressed as Gallic acid equivalents (GAE), based on the calibration curve.

### 2.3. Determination of Antioxidant Capacity

*Principle*. The FRAP (Ferric Reducing Ability of Plasma) assay measures the change in absorbance at 593 nm which owes to the formation of a blue colored Fe^2+^ tripyridyltriazine (TPTZ) compound from the colorless oxidized Fe^3+^ form, by the action of electron donating antioxidants. This method enables an unselective measurement of total antioxidant capacity; this method measures the overall antioxidant capacity and not a specific antioxidant [14]. This measurement enables us to determine the fraction of polyphenols from the total antioxidants.

Reagents are as follows:

 2,4,6-Tri(2-pyridyl)-1,3,5-triazine (TPTZ) (10 mM) in hydrochloric acid (HCl) (40 mM).
 Acetate buffer (300 mM, pH = 3.6).
 FeCl_3_·6H_2_O (20 mM).
 A FRAP reagent, which was prepared by mixing acetate buffer, TPTZ, and FeCl_3_ in the ratio of 10 : 1 : 1.

*Procedure*. 100 *μ*L of seminal plasma was added to 3.0 mL of the freshly prepared FRAP working solution, followed by mixing it uniformly. The absorbance of the solution was measured on a spectrophotometer at 593 nm against a blank which was prepared by adding 1 mL of the FRAP reagent to distilled water and the concentration of the total antioxidant capacity (TAC) was expressed as ascorbic acid equivalents.

Means and standard deviations were calculated to describe quantitative variables. For univariate analysis, *t*-test for independent sample was performed as appropriated. For multivariate analysis, we used multiple linear regressions. *p* values <0.05 determined statistical significance for all analyses.

## 3. Results

A total of 67 semen samples were collected and assessed in 67 IVF fresh cycles during the study period. Patient and semen characteristics are presented in Table 1. In one cycle, no eggs were retrieved; therefore, no embryo transfer was performed.

An association between motility and semen sample volume (*r* = 0.258, *p* = 0.04) as well as sperm concentration (*r* = 0.309, *p* = 0.01) was observed. Antioxidant concentration was significantly associated with sperm concentration and total motile count (TMC) (*r* = 0.352, *p* = 0.008 and *r* = 0.527, *p* < 0.001, resp.). No association was found between polyphenols and any of the evaluated semen parameters (Table 2). Insemination fertilization rate was associated with higher TMC (*p* = 0.005); however, no correlation was found between insemination fertilization rate and antioxidants nor polyphenols concentrations. ICSI fertilization rates were not associated with antioxidants or polyphenols as well.

We analyzed semen concentration according to the updated WHO 2010 semen analysis criteria [15]. Normal sperm concentration considered ≥15 × 10^6^/mL (Table 3). In the group of normal sperm concentration (*n* = 53, group 1), a significantly higher antioxidants concentration was measured compared with the group of sperm concentration < 15 × 10^6^/mL (*n* = 14, group 2) (830.3 ± 350 *μ*M and 268.3 ± 220 *μ*M, resp., *p* < 0.001). Using multiple logistic regression models, controlling for motility and antioxidants levels, the association between concentration to antioxidants and motility remained significant (*p* = 0.004, 0.021, resp.).

Polyphenols concentration did not differ between the groups of sperm concentration above and below 15 × 10^6^ (178.7 ± 121 *μ*M and 161.7 ± 61 *μ*M, resp., *p*-NS). As expected, group 1 had significantly higher sperm motility compared with group 2 (61.7 ± 15% and 43.6 ± 25%, *p* = 0.023).

The semen fluid antioxidants and polyphenols concentrations among the group of patients that had pregnancy did not differ statistically compared with the group of patients who did not conceive.

## 4. Discussion

It is known that reactive oxygen species can cause male subfertility [8, 16–18]. In our prospective study, we have shown that a statistically significant correlation exists between antioxidants concentration in semen fluid and sperm concentration as well as with TMC. A significant higher antioxidants concentration in semen fluid was found in the group of patients that had sperm concentration above 15 × 10^6^. The motility in this group was also significantly higher. We did not find any correlation between pregnancy rates via IVF insemination neither via ICSI insemination. In this study, we measured polyphenols concentration in addition to antioxidants concentration. Although polyphenols are one of the abundant antioxidants in nature, as far as we know there is no information in the literature regarding polyphenols in semen fluid. Our purpose was to characterize levels of polyphenols in semen fluid and to understand the correlation to sperm characteristics and fertility treatment results. As being part of total antioxidants content, the concentration of polyphenols was lower than the total antioxidants concentration. However, while total antioxidants concentration was significantly higher in the group of sperm concentration ≥15 × 10^6^, the polyphenols concentration did not differ between the groups of sperm concentration lower and above 15 × 10^6^. A possible explanation for this observation could be that polyphenols have synergistic effect with other active antioxidants that we did not measure specifically, but their presence was expressed in the total antioxidants concentration. Polyphenols concentration did not correlate with pregnancy rates in IVF insemination nor in ICSI insemination.

In conclusion, this study shows that antioxidants concentration correlates with sperm concentration and motility. This study is the first to investigate polyphenols concentration in semen fluid. Although polyphenols concentration was found in significant amount within semen fluid, it did not correlate with any of the measured parameters. More studies are needed in order to investigate polyphenols role in male fertility.

## Figures and Tables

**Table 1:** Patients, semen, and cycle characteristics.

Paternal age average (years ± SD)	36.7 ± 9.9
Maternal age average (years ± SD)	32.0 ± 5.3
Ethnicity	
Jewish (%)	35.4
Muslim (%)	64.6
Semen volume (mL ± SD)	2.9 ± 1.7
Semen concentration (×10^6^/mL ± SD)	47.7 ± 44.9
Sperm motility (% ± SD)	58.5 ± 19.3
Retrieved eggs (number ± SD)	9.2 ± 4.8
Transferred embryos (number ± SD)	1.6 ± 0.6
Cycle outcome	
Chemical pregnancy	5
Normal clinical pregnancy	16
Blighted ovum	3
Dead fetus	2

**Table 2:** Correlations between sperm parameters, antioxidants, polyphenols, and treatment success.

	Volume		Concentration		Motility		Antioxidant		Polyphenols		Paternal age		Embryos transferred		Total motility	
	*r*	*p*	*r*	*p*	*r*	*p*	*r*	*p*	*r*	*p*	*r*	*p*	*r*	*p*	*r*	*p*
Concentration	0.012	0.92														
Motility	0.258	0.04	0.309	0.01												
Antioxidants	0.078	0.57	0.352	0.008	0.041	0.76										
Polyphenols	0.101	0.44	0.015	0.90	0.021	0.87	0.049	0.72								
Paternal age	0.128	0.32	0.146	0.25	0.066	0.60	0.156	0.25	0.044	0.741						
Et embryos	0.101	0.44	0.237	0.07	0.03	0.82	0.078	0.58	0.098	0.467	0.377	0.003				
Total motile	0.537	<0.001	0.882	<0.001	0.354	0.05	0.527	<0.001	0.038	0.77	0.02	0.87	0.236	0.07		
ICSI fertilization	0.187	0.16	0.179	0.18	0.101	0.46	0.040	0.782	0.005	0.972	0.049	0.72	0.053	0.705	0.186	0.171
Ins. fertilization	0.094	0.633	0.166	0.39	0.064	0.74	0.081	0.68	0.255	0.19	0.404	0.03	0.081	0.69	0.053	0.814

**Table 3:** The association between the evaluated parameters and semen concentration according to WHO criteria 2010.

	Concentration ≥ 15 (*n* = 53)	Concentration < 15 (*n* = 14)	*p*
Volume (mL ± SD)	2.9 ± 1.8	3.0 ± 1.2	0.846
Motility (% ± SD)	61.7 ± 15	43.6 ± 25	0.023
Antioxidant (*μ*M ± SD)	830.3 ± 350	268.3 ± 220	<0.001
Polyphenols (*μ*M ± SD)	178.7 ± 121	161.7 ± 61	0.487
Paternal age (years ± SD)	37.4 ± 10.5	34.0 ± 7.5	0.191
Transferred embryos (number ± SD)	1.6 ± 0.6	1.5 ± 0.7	0.612
